# Factors that influence the scope of practice of the chiropractic profession in Australia: a thematic analysis

**DOI:** 10.1186/s12998-024-00535-2

**Published:** 2024-05-27

**Authors:** Desmond Wiggins, Aron Downie, Roger Engel, Sandra Grace, Benjamin T. Brown

**Affiliations:** 1 Department of Chiropractic, Macquarie University, Sydney, Australia; 2 Faculty of Health, Southern Cross University, Lismore, Australia

**Keywords:** Australia, Chiropractic, Scope of practice, Thematic analysis

## Abstract

**Abstract** Scope of practice has been defined as the activities that an individual health care practitioner is allowed to undertake within a specific profession. The chiropractic profession in Australia does not currently have a documented scope of practice. Informed discussions around scope of practice are hampered by a paucity of literature in this area. Acknowledging this void in the literature, we chose to investigate the factors that influence scope of practice of the chiropractic profession. A knowledge of the factors will facilitate discussion on the topic and help the profession to work towards establishing a scope of practice.

**Aim** The aim of this study was to identify the factors that influence scope of practice of chiropractic in Australia from the perspective of 4 stakeholder groups within the profession.

**Methods** This study employed semi-structured, online-interviews. Open-ended questions, guided by a flexible interview protocol, and augmented by supplemental questions, probes and comments, were used to gather data on the research question. Data were analysed using reflexive thematic analysis.

**Results** Six factors that influenced scope of practice of chiropractic were identified in this study: education, evidence (research-derived and practice-based), political influence, community expectations, entrepreneurial business models, and geographical location.

**Conclusion** Knowledge of the factors that influence scope of practice of chiropractic in Australia is important for establishing a scope of practice for the profession. This knowledge is also of value to a range of stakeholders including patients, health care providers (within and outside the profession), professional associations, and policymakers.

**Supplementary Information:**

The online version contains supplementary material available at 10.1186/s12998-024-00535-2.

## Introduction

It is important to acknowledge that there are several, often heterogeneous, definitions of scope of practice (SOP) in the literature, and that these definitions may refer to either the SOP of a profession or an individual practitioner. Given the lack of literature on the SOP of the chiropractic profession in Australia, a working definition for SOP is the 'full spectrum of roles, functions, responsibilities, activities and decision-making capacity that individuals within that profession are educated, competent and authorised to perform’ [1]. The lack of consensus on what defines SOP for a health care profession has contributed to uncertainty, confusion, and professional conflicts (inter- and intra-professional) regarding what constitutes SOP [2].

While it has been accepted that SOP for a profession is difficult to define, [2–4] a definition of health professions’ SOP is important as it forms the base from which governing bodies formulate standards of practice, educational institutions prepare curricula, and employers construct job descriptions [2]. More importantly, if a health care profession has a defined SOP, and the practitioners within the profession abide by the SOP, it potentially improves patient safety [5]. This is also true for chiropractic in Australia.

Within the Australian context only 1 out of 16 regulated health professions in Australia (dental) has a defined, accessible, and documented SOP for the profession [6, 7]. This scenario has resulted in SOP being implied, or assumed by stakeholders internal and/or external to a health care profession. Evidence for this is seen in the Australian Federal Health Minister’s recent suggestion (2023) that allied health professions (which includes chiropractic) should be able to ‘expand their scope of practice’ as part of the proposed changes to *Medicare*, the insurance scheme that provides Australian citizens and permanent residents access to health and hospital services at zero or relatively low cost [8, 9].

As the vast majority of regulated health professions in Australia, including chiropractic, do not have a defined SOP, requests for a profession to expand or curtail their SOP (such as the Australian Federal Health Minister’s recent proposal) pre-supposes that a well-defined SOP already exists for that profession.

### Aim

The aim of this study was to identify the factors that serve either as a barrier to or enabler of the SOP of chiropractic in Australia from the perspective of 4 stakeholder groups within the profession (*i.e.* clinical educators, professional associations, regulatory body, and the educational accreditation body). Knowing these factors will help close the gap on the topic of SOP, will help facilitate discussion on SOP of chiropractic in Australia, and bring the profession one step closer to being able to build a framework for developing a SOP.

## Methods

### Eligibility and recruitment

This study engaged with 4 chiropractic stakeholder groups which were comprised of 8 representative bodies (Table 1). We employed a purposive sampling strategy to recruit participants. This strategy was used to help ensure appropriate breadth in participant characteristics and to capture a broad range of ideas on the topic. The stakeholder groups were contacted via email by a member of the research team (AD) who requested help in recruiting 3 representatives from each group. Importantly, the final sample size was not decided *a priori*. Rather, the study sought for a representative selection of 3 members from each of the 8 representative bodies (total: 24 participants), which was considered sufficient to achieve data saturation [10–14]. If saturation was not achieved, further participants would be recruited.

**Table 1:** Four chiropractic stakeholder groups

Stakeholder groups (*n* = 4)	Representative bodies (*n* = 8) within stakeholder groups
Regulatory Board	Chiropractic Board of Australia (CBA)
Educational Accreditation Body	Council on Chiropractic Education Australasia (CCEA)
Professional Associations	Australian Chiropractor’s Association (ACA)
	Chiropractic Australia (CA)
Clinical Educators	Central Queensland University (CQU)
	Macquarie University (MQ)
	Murdoch University (MU)
	Royal Melbourne Institute of Technology University (RMIT)

Participants were included in this study if they were associated with one of the 8 representative bodies in Australian chiropractic as either: a board member of the Australian Chiropractors Association and/or Chiropractic Australia (the professional associations which represent the majority of registered chiropractors); or the Chiropractic Board of Australia (representing the body that sets regulation standards); or a member of the Council on Chiropractic Education Australasia (representing the accreditation authority responsible for accrediting education providers and programs of study for the chiropractic profession); or were a clinical educator within the chiropractic department of Central Queensland University, Macquarie University, Murdoch University, and Royal Melbourne Institute of Technology University (representing the accredited Australian universities). Additionally, participants had to be willing to undertake a 30–45 min online interview regarding their understanding of the factors that influence SOP of the chiropractic profession in Australia to be included.

In order to attempt to protect anonymity, participant data were de-identified prior to analysis by removing all reference to name, professional role, professional affiliation, geographical location, employment, and education. The research topic and the associated questions were not discussed with participants prior to the interview. None of the participants were known to the interviewer prior to being interviewed. No financial compensation was offered for participation.

### Data collection

Online, semi-structured interviews which relied on asking questions within a predetermined thematic framework were conducted between the 9th of December 2022 and the 3.^rd^ of March, 2023 by DW. When conducting these interviews, DW loosely adhered to the interview agenda. Discussions were guided by what DW interpreted to be meaningful to the interviewee, and would often weave in and out of different topics using open-ended questions, (Additional file 1) augmented by supplemental questions, probes and comments. This approach allowed participants, rather than the interviewer, to direct the discussion and encouraged participants to share their personal experiences, attitudes, perceptions and beliefs about the topic [15].

Questions were partly informed by 2 recent studies that looked at the factors that influence SOP in Australia. One study investigated the factors that influenced SOP of chiropractic, (6) while the other focussed on the factors that influenced SOP of the 5 largest regulated health professions (nursing, medical practice, pharmacy, physiotherapy, and psychology). (7) Four factors were discovered that influenced chiropractic SOP; education, professional identity, organisational structure, and patient safety, while 8 factors influenced the SOP of the 5 largest regulated health professions; education, competency, professional identity, role confusion, legislation and regulatory policies, organisational structures, financial factors, and professional and personal factors.

The conversational style of interaction between interviewer and participants helped reduce the potential for a rapid exchange of questions and/or one-line responses. Prior to the start of data collection, the questionnaire and interview process were piloted on a chiropractic clinical educator not associated with the study. The interview was reviewed by an experienced qualitative researcher (SG) as well as a member of the research team (BB). All interviews were conducted by DW using Microsoft Teams^©^ (Redmond, WA, USA). A reflexivity statement for DW has been provided in Additional file 2.

### Ethics approval

Ethics approval for the study was granted by Macquarie University’s Human Research Ethics Committee (Reference No: 520221203941883). This study has been reported in accordance with the Consolidated Criteria for Reporting Qualitative Research (COREQ) [16].

### Data analysis

Data were analysed using reflexive thematic analysis from an interpretivist perspective. Thematic analysis is a commonly used method for analysing qualitative data to identify, analyse and interpret meaning through a systematic process of generating codes that leads to the development of themes. This approach provided a flexible method for developing, analysing and interpreting patterns across the data which was important to the study [17]. Following transcription and data cleaning of the audio recordings, participants were given an opportunity to review and make comment on their interview transcript. After review, the recorded audio file and transcription were de-identified. Data were stored on a secure cloud-based storage system at Macquarie University (Microsoft SharePoint^©^,Redmond, WA, USA).

Prior to the initial round of coding, ‘pre-coding’ (highlighting significant participant quotes or passages worthy of attention) [18] was conducted by DW. A total of 22 coding rounds were undertaken by DW with the first round conducted on 9 March, 2023. Codes were discussed with BB and refined, coalesced and expanded as required. Subsequent coding and theme development were discussed fortnightly with BB, AR, and RE. In the final round on 28 April, 2023, themes were agreed by all authors.

A ‘code’ was typically either a phrase or paragraph that reflected relevant information about barriers to, or enablers of, SOP. Importantly, codes and themes were not determined *a priori*, rather they were developed as part of the data analysis process. The coding process was conducted using NVivo.^©^ (version 20, Lumivero, Denver, CO, USA). The analysis was guided by the 6-step framework for conducting thematic analysis as described by Braun and Clarke; familiarisation of data, generation of codes, combining codes into themes, reviewing themes, determining significance of themes, and reporting findings [19].

## Results

**Fig. 1:**
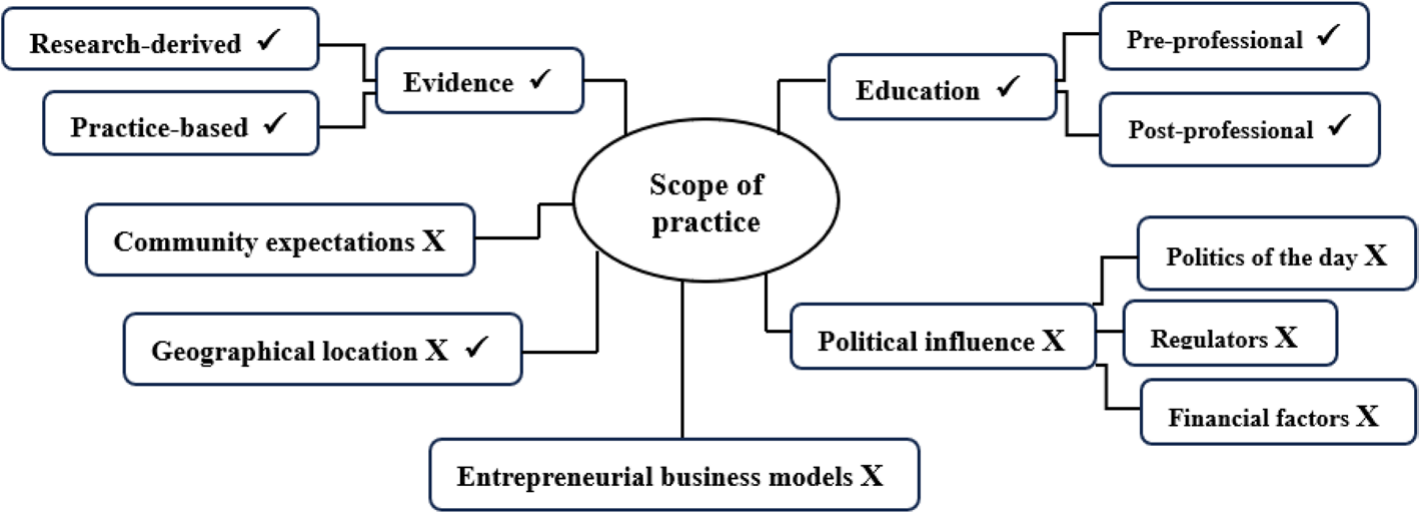
Factors that influence SOP of chiropractic in Australia. Legend:  ✔ =  Enabler of SOP; X = Barrier to SOP; X  ✔ = Both barrier to, and enabler of, SOP

One representative body opted to not participate in the study. As a result, 7 representative bodies, comprising 21 participants, were involved in the study. No new information was forthcoming at interview 18, suggesting that data saturation had been achieved. To confirm this, 3 additional interviews were undertaken, which did not uncover additional themes. Six themes (listed here in order of importance to participants) were generated from the data analysis process: education; evidence (research-derived and practice-based); political influence; community expectations; entrepreneurial business models; and geographical location. Figure 1 illustrates the discovered factors and identifies if they were a barrier to, or an enabler of, SOP.

### Theme 1: education

Participants expressed the opinion that pre- and post-professional education were focal elements that were an enabler of SOP of chiropractic in Australia.

#### Pre-professional education

The vast majority of participants (19/21) indicated that pre-professional education (*i.e.* undergraduate studies) was an enabler to SOP as it provided the base knowledge about the profession. However, several participants expressed frustration with a perceived lack of comparable pre-professional education across the accredited chiropractic programs. They commented on observed differences in SOP within various sections of the profession depending on where chiropractors were educated. For example:

When I'm talking about scope of practice, it could initially be at an undergraduate level. Their undergraduate education would give them foundational knowledge in [the] field. (P7)

I think we have a scope of practice, but it varies depending on which institution you've come from. (P21)

#### Post-professional education

A large number of participants (18/21) highlighted that post-professional education (*e.g.* post-graduate university studies and/or continuing professional development [CPD] programs) was an enabler of an expanded SOP. This viewpoint was based on the idea that post-professional education increases the knowledge and skill-base of a practitioner to a level over and above that which is recognised as a normal SOP [20]. Participants emphasised that post-professional or CPD programs needed to comply with Australian educational standards and be accredited by the regulatory authorities. They felt such an approach was essential to protect the public, regulate the profession, expand SOP, and help determine educational standards. For example:

*Postgraduate offerings [such as] CPD result in a specialist scope of practice once you enter into postgraduate education [but] postgraduate education needs to be accredited. (P7)*

*Other things that determine our scope of practice would be the types of further education that are available to us in the Australian context of education. (P16)*

### Theme 2: evidence

An evidence-informed approach to health care has been defined as the combination of three separate, yet inter-related components; the best available clinical evidence from systematic research, individual practice-based evidence, and patient values or preferences [21–24]. Commentary was made by participants regarding the first two components, but not the third.

#### Research-derived evidence

Several participants (14/21) proposed that research-derived evidence was an enabler of SOP as it had the potential to inform personal, professional, and legislative scopes of practice. Additionally, participants indicated that this form of evidence influenced SOP at a more rapid rate than the training provided by educational providers, as institutions typically take time to modify their teaching programs. For example:

*We’re always exploring evidence in a way that we can continue to evolve what we've learned, because obviously universities take a while to modify what they're doing sometimes. Understanding [scientific] evidence [helps] you derive different ways of practicing. (P17)*

#### Practice-based evidence

Some participants (11/21) were concerned that practice-based evidence was often overlooked as a legitimate enabler of the profession’s SOP, while others acknowledged that the combination of scientific and practice-based evidence was an enabler of SOP. For example:

*I've defined scope of practice based upon research that I've undertaken, but not just evidence, also my personal experience [as a chiropractor]. (P7)*

*Scope [of practice] is affected also by your own experience in clinical practice and understanding - what you know and you see that works. (P17)*

### Theme 3: political influence

A large number of participants (15/21) reported that political influence (*i.e.* agents working within the political system making official government decisions in a given period) had been a barrier to SOP of chiropractic through various means such as politics of the day, controls established by government regulators, government intervention, and restrictions on practice due to financial factors related to government reimbursements.

#### Politics of the day

Several participants (9/21) suggested that politics of the day (*i.e.* the political system making official government decisions in a given period) had at times acted as a barrier to SOP by limiting the SOP of chiropractic. This perspective was based on the ban placed on spinal manipulation of children under the age of 2 years implemented in 2019 by a state government [25]. This prohibition arose when Safer Care Victoria, acting on the direction of the Victorian Minister for Health and the Council of Australian Governments (COAG) Health Council (CHC), undertook an independent review of the practice of chiropractic spinal manipulation on children under 12 years [25]. For example:

*I think politics is a barrier [to scope of practice]. You don't have to look much further than the Safer Care, Victoria [case] to realise that politics can at times have a great influence on what we do [scope of practice]. (P19)*

#### Regulators

Several participants (14/21) expressed the opinion that the chiropractic regulators (Australian Health Practitioner Regulation Agency [Ahpra] and Chiropractic Board of Australia [CBA]) had acted as a barrier to SOP by generating a SOP without the input of the profession. This notion incorrectly stemmed from respondents' confusing the profession's current code of conduct guidelines with a SOP document For example:

Our scope of practice is defined by our National Board [CBA] with influence from Ahpra. (P19)

Well, I mean that there's one [SOP document] that we can read from Ahpra…. It must have been in the Code of Conduct [document]. (P1)

#### Financial factors

A number of participants (13/21) felt that financial decisions made by the government such as reducing the *Medicare* remuneration rates for chiropractic services and allowing overseas third-party insurers to dictate private insurance rebates for chiropractic were a barrier to SOP. For example:

*There's probably pressure to limit chiropractic scope of practice indirectly, I'd say through reduction in Medicare rebates. (P15)*

*We're seeing overseas insurance companies [who] are third payers actually driving scope [of practice]as well. (P20)*

*Insurance companies are allocating less funds for patients to attend chiros. (P2)*

### Theme 4: community expectations

Numerous participants (13/21) expressed the opinion that community expectations around SOP of chiropractic and what a chiropractor ‘is and/or does’, had for the most part been a barrier to SOP through the use of internet content and social media platforms such as Facebook and/or Twitter.

#### Social media

Although accessing social media to share views, information, and experiences has become common place for most Australians, [26] some participants (9/21) considered that social media had been a barrier to chiropractic as it confused public perception about what the SOP was. For example:

*I think you can't discount social media [as] there's just so many social media groups out there now within chiropractic [and] outside of chiropractic that are moulding the direction [of the profession]. I think it's difficult for the public, to know what it is they're going to get [from a chiropractor]. They can go to [chiropractic] websites but the standards of care [on the sites] aren’t universal and have driven our change in agenda and our scope [of practice].(P20)*

### Theme 5: entrepreneurial business models

Several participants (9/21) expressed their concern that entrepreneurial business models had been a barrier to SOP by negatively influencing members of the profession. These models were promoted as a way of maximising clinical income with reduced treatment times and limited depth and breadth of information that was provided to patients. For example:

*There are entrepreneurial groups that have got very little research that are selling all sorts of stuff to practitioners which is quite frankly inappropriate. (P13)*

*Students are under enormous financial pressure. I think they come out with quite big HECS* [Higher Education Contribution Scheme] *fees and our cost of living is pretty high. I think all of that does make a certain [entrepreneurial] business model popular. (P11)*

### Theme 6: geographical location

Several participants (10/21) identified geographical location, particularly rural and remote areas, as both a barrier to, and an enabler of, SOP. For example, participants felt that limited access to medical and/or other allied health professionals in rural and remote areas acted as a barrier to the proper function of SOP as some chiropractors may have been induced to work outside their normal SOP. Conversely, some participants suggested that the unequal distribution of health professionals had at times acted as an enabler to SOP as some chiropractors had undertaken post-professional education to expand their SOP in order to meet the needs of the community. This expanded SOP could involve a range of clinical practices that were not typically considered part of chiropractic treatment (*e.g.* prescribing rights and advanced imaging).

For example:

*I think because of isolation and [lack of] availability of [other] health care in remote areas, people rely on any professional to do more or to push slightly outside their scope [of practice] because they just don't have the availability of other healthcare facilities. (P21)*

## Discussion

This is the first study to explicitly explore the factors that influence SOP of chiropractic in Australia from the perspective of representatives of 4 stakeholders within the profession. Six themes were identified which represented factors that influenced SOP of chiropractic: education; evidence (research-derived and practice-based); political influence; community expectations; entrepreneurial business models; and geographical location.

### Education

Both pre- and post-professional education were identified as significant enablers of SOP of chiropractic. This notion was consistent with the literature around SOP of regulated health professions in Australia. For example, Scanlon *et.al.* [27]*,* and Birks *et.al* [28]*.* reported that organised and available education was a natural enabler to advanced SOP. Similarly, Duffield *et.al* [29]*.* highlighted that post-professional education was an enabler to an expanded SOP by generating a greater depth and breadth of knowledge and clinical skills, and enhanced higher data synthesis capability. Additionally, practitioners who had undertaken post-professional education were more likely to be involved in scientific research [29]. Similarly, de Luca *et.al* [30]*.* and Innes *et.al* [31]*.* found that education was an enabler of SOP as it informed professional identity of chiropractic in Australia.

Some participants commented that SOP was dependent on where a chiropractor had undertaken their pre-professional education. This viewpoint is supported in the literature by de Luca *et.al* [30]*.* who administered a cross-sectional survey to Australian and New Zealand chiropractic students to explore their viewpoints on SOP, professional identity, and future chiropractic practice as it related to chiropractic education. These authors reported that the chiropractic institution attended by a student accounted for over 50% of the variance of student opinions around SOP, a scenario that may have created a heterogeneity between chiropractic schools and an inconsistent SOP across the profession [30].

### Evidence: research-derived and practice-based

The view that research-derived evidence was particularly relevant to SOP is supported by government publications that show 4 out of 10 patients regularly received care that was ineffective, unnecessary, or potentially harmful as treatments were often not based on current evidence [32]. Studies have shown that research-derived evidence is an enabler of SOP as it improves patient safety as well as clinical outcomes and reduces health care costs [33, 34]. Similarly, Benton *et.al* [35]*.* and Watkins *et.al* [36]*.* found that if SOP is evidence informed patient safety is preserved and outcomes are optimised. The Royal Children's Hospital Centre for Community Health, [37] the Oxford Review, [38] and Wilkinson [21] highlight that practice-based evidence is an enabler of SOP because when this type of evidence is integrated into clinical practice the individual risks and benefits of potential interventions can be assessed which leads to an improvement in patient outcomes.

Some participants felt that educational facilities are much slower than primary care settings to implement evidence. This notion has support from Scott [39] who emphasised that the implementation of a new evidence-based paradigm typically takes 2–4 years to become embedded in educational facilities. Similarly, Soicher *et.al* [40]*.* highlight that evidence-based practices are often slow to be instituted into college classrooms. Slow implementation of evidence into practice hinders the expansion of SOP, which may in turn negatively impact outcomes and quality of life for patients, productivity, and healthcare costs [32].

### Political influence

Political influence has typically been thought of as only involving national governments, [41] but Salvage *et.al* [42]*.* emphasise that political influence can act as a barrier to SOP of health care professions at all levels including local, regional, and national governments. The recent limits imposed on clinical practice by governments in some Australian states during the COVID-19 pandemic are an example of the impact of political influence on SOP [1]. The impact of political influence on SOP has been acknowledged by sections within the Australian tertiary education sector. For example, the University of Newcastle developed a module in its nursing degree dedicated to exploring the impact of political influence on SOP [43].

An important and unexpected finding in regard to the regulator’s role in SOP was that participants typically expressed the opinion that the regulators had defined and/or documented the SOP for the profession. However, the regulatory bodies do not currently play a role in defining or documenting the SOP of chiropractic in Australia. [6, 44] Interestingly, although there has been a modest increase in the *Medicare* rebate for chiropractic services between 2005 and 2023 [45, 46] some participants incorrectly held the view that the federal government had reduced *Medicare* rebates for chiropractic services.

### Community expectations

Several participants voiced concerns that the influence of social media on community expectations had the potential to be a barrier to SOP in that public safety may be jeopardised if practitioners use social media platforms like Facebook and/or Twitter in an inappropriate manner *e.g.* making non-evidence-based claims about the efficacy of a particular treatment. Participants pointed out that this type of behaviour had already resulted in an action by the officials against the whole profession in restricting the practice of chiropractors in the paediatric field [25].

This view is supported in the literature by Hao *et.al* [47]*.* who assert that an incorrect use of social media by health care professionals can violate a profession’s regulations and may inadvertently cause potential harm to patients. Petrie *et.al* [48]*.* and Scruth *et.al*. [49] caution practitioners to be aware that information they share on social media platforms is considered public by those who view it. This has the potential to create liability at the highest level of a healthcare system. Social media content not only reflects practitioners’ professionalism, but also has the capacity to influence public opinion of the profession. Additionally, acting inappropriately on social media platforms could result in punitive action by the regulators including the cancellation of a practitioner’s registration [49].

### Entrepreneurial business models

Over the past few years, healthcare has experienced significant expansion, becoming the fastest-growing sector in large economies like the US [50–52]. The George Washington University School of Business claimed in 2022, that entrepreneurship and health care practices are related because entrepreneurial business models are currently driving the innovation and technology of the healthcare industry, a scenario that has the potential to interfere with traditional health care [53].

Although entrepreneurial business models in health care seek to ‘address social needs and create a more just equilibrium rather than personal profit’, [54] some participants expressed the opinion that entrepreneurial business models within Australian chiropractic were a barrier to SOP as these models were geared toward personal (practitioner) gain rather than the creation of a just equilibrium. They described how a growing number of chiropractors were using unethical entrepreneurial business models to substantially increase revenue, an approach that has been reported in the literature as being potentially detrimental to patient care [55, 56]. Others highlighted that while disagreeing with certain entrepreneurial business models, they acknowledged that the substantial cost associated with pre-professional chiropractic training made these models of practice very enticing to new graduates who may have accrued substantial debt as part of their chiropractic training.

### Geographical location

Numerous authors reported that a lack of referral options in rural and remote settings had been both a barrier to, and an enabler of, SOP. For example, in these settings health professionals often work outside their SOP due to the lack of other health professionals to refer to [57–63]. Participants also recognised that when compared with their metropolitan counterparts, health practitioners in rural and remote locations were often called upon to provide a wider range of clinical expertise than that required by health professionals practising in metropolitan locations. This scenario has acted as a barrier to SOP as it had been associated with higher levels of practice dissatisfaction in rural and remote regions as individuals in these regions have felt inadequately prepared for the extra responsibilities and technical challenges associated with an expanded SOP [64].

Other barriers to SOP of practitioners in rural and remote locations include minimal access to professional development and a lack of peer support and difficulties associated with fostering inter-professional teamwork [65]. An international study investigating rural and remote practices across 5 countries found that practising in rural and remote areas posed a significant barrier to SOP due to obstacles such as excessive travel and a disproportionate workload [66]. This viewpoint was based on the knowledge that rural practitioners encountered challenges due to travel distances and excessive workloads [66]. Conversely, rural and remote settings can act as an enabler of SOP as many health care professionals undertake further education to expand their SOP in order to meet community needs [67]. The development of an expanded SOP allows interested practitioners to broaden their knowledge, skills and training in order to acquire a unique professional qualification for their discipline. With an extended SOP such practitioners become valued assets to an otherwise understaffed healthcare system due to the broader range of services they can provide [68].

### Strengths and limitations

There are several strengths of this study. The use of semi-structured interviews allowed for the guidance and redirection of questions as necessary in real-time. This meant that the research framework could be easily revised as new information arose. The research process was not limited to the issues that the researcher may have considered important; rather it uncovered issues that participants considered were essential. The use of reflexive thematic analysis from an interpretivist approach, rather than a positivistic approach that focusses on exact understanding and meaning [69, 70], allowed codes and themes to evolve during the interview process. The absence of data on participant characteristics ensured participant anonymity and allowed participants to express their opinions openly without concern that their opinion would be construed as the view of their stakeholder group.

It is possible that increasing the number of participants from each stakeholder group may have altered the breadth and depth of responses. However, in qualitative research, interviews usually continue until no new information is gained from further interviews (theoretical saturation). Given that no new information was forthcoming at interview 18, we believed that saturation had been reached. However, we undertook 3 more interviews to confirm saturation. No new themes were revealed which confirmed saturation.

## Conclusion

This study investigated the factors that influence scope of practice of chiropractic in Australia from the perspective of 4 stakeholders within the profession *i.e.* clinical educators, professional associations, regulatory body, and the educational accreditation body. Six themes were identified. The results show that the representatives of the 4 stakeholder groups perceived that scope of practice was influenced by a variety of factors; education (pre- and post- professional), political influence, community expectations, evidence (research-derived and practice-based), entrepreneurial business models, and geographical location. Knowing these factors will help facilitate discussion on SOP of chiropractic in Australia, close the gap in the literature on the topic of SOP, and bring the profession one step closer to being able to build a framework for developing an SOP. This has the potential to lead to a definition of scope of practice of chiropractic that is broadly accepted by the profession. Although this study focused on the SOP of chiropractic in Australia it should be acknowledged that our findings may have relevance to other jurisdictions that have a national licensing system for chiropractic *e.g.* New Zealand.

### Supplementary Information

**Additional file 1: Appendix 1. **Questionnaire for interviews.

**Additional file 2: Appendix 2. **Reflexivity statement - Desmond Wiggins.

## Data Availability

All data generated or analysed are included in this published article. The participants of this study did not give written consent for their data to be shared publicly, so the supporting data cannot be shared openly.

## Abbreviations

ABS: Australian Bureau of Statistics
ACA: Australian Chiropractor’s Association
Ahpra: Australian Health Practitioner Regulation Agency
AMAQ: Australian Medical Association Queensland
CA: Chiropractic Australia
CBA: Chiropractic Board of Australia
CCEA: Council on Chiropractic Education Australasia
CQU: Central Queensland University
(COREQ): Consolidated criteria for reporting qualitative research
CPD: Continuing professional development
HECS: Higher Education Contribution Scheme
MQ: Macquarie University
MU: Murdoch University
PICF: Participant information consent form
RMIT: Royal Melbourne Institute of Technology University
SOP: Scope of practice
