# Supplementary material for: Factors that influence the scope of practice of the chiropractic profession in Australia: a thematic analysis
Source: Chiropr Man Therap. 2024 May 27;32:18. doi: 10.1186/s12998-024-00535-2 (PMC11131270; doi:10.1186/s12998-024-00535-2)
Supplement: Supplementary file 1 — Additional file 1: Appendix 1. Questionnaire for interviews. [file 12998_2024_535_MOESM1_ESM.docx]

**Appendix 1: Questionnaire for interviews**

1. What is your understanding of the scope of practice for a profession?
2. Do you think the chiropractic profession in Australia has a defined scope of practice?
3. Do you think other health professions have a scope of practice?
4. What do you think influences, or could influence, scope of practice of the Australian chiropractic profession?
5. Do you think that scope of practice for the profession is evolving?

6. Do you think there is a difference between the personal, professional and

legislative scope of practice?

1. Case illustration 1: Physiotherapists administering vaccinations and having limited prescribing

rights. Is administering vaccinations and/or prescribing anti-inflammatories potentially part of scope of practice of chiropractors in Australia?

8. Case illustration 2: Chiropractors in Oregon (US) can practice obstetrics, perform minor

surgery and proctology after completing post-professional training. Could this be part

of scope of practice of chiropractic in Australia?
