# Supplementary material for: Factors that influence the scope of practice of the chiropractic profession in Australia: a thematic analysis
Source: Chiropr Man Therap. 2024 May 27;32:18. doi: 10.1186/s12998-024-00535-2 (PMC11131270; doi:10.1186/s12998-024-00535-2)
Supplement: Supplementary file 2 — Additional file 2: Appendix 2. Reflexivity statement - Desmond Wiggins. [file 12998_2024_535_MOESM2_ESM.docx]

**Appendix 2: Reflexivity statement - Desmond Wiggins**

I am a 71-year-old married, white male who has a 51-year-old son. I have practiced as a chiropractor for forty-five years in Queensland and New South Wales. I grew up in mostly urban areas of Queensland and currently live in a small rural town in Queensland. I undertook my chiropractic training between 1976 and 1978 in a private college located on the Sunshine Coast of Queensland. At that time this was a common way to become a chiropractor. On reflection, I realise that understanding the scope of practice of chiropractic was not a requirement of my training. My first in-depth experience with ‘scope of practice of chiropractic’ was when I explored the 19^th^ century origins of chiropractic as part of my Master of Research studies at Macquarie University between 2016 and 2020. Prior to that, I had no knowledge of the factors that influenced scope of practice of chiropractic in Australia nor did I have any pre-conceived ideas about what comprised scope of practice. I decided that I would like to study scope of practice of the profession in greater depth but was disappointed by the lack of information available in the literature to help guide my thinking. I am currently undertaking Doctor of Philosophy studies in the Chiropractic Department at Macquarie University. My studies have influenced my opinion that the chiropractic profession in Australia should have a well-defined and documented scope of practice.

Therefore, I undertook this research to discover the factors that influence scope of practice of chiropractic in Australia in an attempt to lay the groundwork for the future formulation of a scope of practice for the chiropractic profession in Australia. Given that I have had no former experience or training in qualitative research and had a limited understanding of the factors that influence the Australian profession’s scope of practice prior to 2021, I believe my past experience in the profession has had little influence on how I have interpreted the research findings.
